# *MTHFR* Gene C677T Polymorphism in Autism Spectrum Disorders

**DOI:** 10.1155/2014/698574

**Published:** 2014-11-06

**Authors:** Elif Funda Sener, Didem Behice Oztop, Yusuf Ozkul

**Affiliations:** ^1^Department of Medical Biology, Erciyes University Medical School, 38039 Kayseri, Turkey; ^2^Department of Child Psychiatry, Erciyes University Medical School, 38039 Kayseri, Turkey; ^3^Department of Medical Genetics, Erciyes University Medical School, 38039 Kayseri, Turkey

## Abstract

*Aim*. Autism is a subgroup of autism spectrum disorders, classified as a heterogeneous neurodevelopmental disorder and symptoms occur in the first three years of life. The etiology of autism is largely unknown, but it has been accepted that genetic and environmental factors may both be responsible for the disease. Recent studies have revealed that the genes involved in the folate/homocysteine pathway may be risk factors for autistic children. In particular, C677T polymorphism in the *MTHFR* gene as a possible risk factor for autism is still controversial. We aimed to investigate the possible effect of C677T polymorphism in a Turkish cohort. *Methods*. Autism patients were diagnosed by child psychiatrists according to DSM-IV and DSM-V criteria. A total of 98 children diagnosed as autistic and 70 age and sex-matched children who are nonautistic were tested for C677T polymorphism. This polymorphism was studied by using polymerase chain reaction-restriction fragment length polymorphism (PCR-RFLP) methods. *Results*. *MTHFR* 677T-allele frequency was found to be higher in autistic children compared with nonautistic children (29% versus 24%), but it was not found statistically significant. *Conclusions*. We conclude that other *MTHFR* polymorphisms such as A1298C or other folate/homocysteine pathway genes may be studied to show their possible role in autism.

## 1. Introduction

Autism spectrum disorders (ASDs) include autistic disorder (or classic autism), Asperger syndrome, pervasive developmental disorders-not otherwise specified (PDD-NOS), and childhood disintegrative disorder. As a childhood disease, autism is characterized by three core symptoms with impaired reciprocal social interaction and communication, a pattern of repetitive behavior and/or restricted interests [1–4]. Clinical diagnosis of ASD is based on behavioral history and behavioral assessments of the clinical features by child psychiatrists. Clinicians usually use validated diagnostics tools. Much more attention has been given to the significant increase in the reported incidence of ASDs in the world today [5]. The rise of autism has been attributed to the changes in diagnostic tools, professional awareness of the disease, and broader diagnostic definition [5, 6]. Today, the prevalence of the disease is reported to be approximately 1/80–100 [4, 5, 7]. ASDs are accepted as multifactorial inheritance with 90% genetic background [5, 8]. As a complex neurodevelopmental disorder, the phenotype and severity of autism are extremely heterogeneous with differences from one patient to another [6, 8, 9]. This heterogeneity involves both locus and allelic heterogeneity in ASD cases [10].

Despite the lack of success in identifying the candidate genes which are responsible for the majority of ASD cases, epigenetic modifications of genes important for normal brain development and growth and cognitive function and behavior are involved in the etiology of ASDs. The term epigenetics means reversible heritable changes in gene expression regulation by modulating rather than by changes in the nucleotide sequence of a gene [7]. Abnormal methylation patterns as epigenetic defects have been implicated in idiopathic ASDs [11, 12] as well as ASD-associated syndromes such as Rett's syndrome, Angelman syndrome, Prader-Willi syndrome, and Fragile-X syndrome [12, 13].

In recent years, some studies have revealed that polymorphisms of the genes are involved in the folate/homocysteine pathway as risk factors for autistic children [14–17]. There is a great deal of evidence suggesting that DNA methylation defects are associated with ASDs, and the role of the methylenetetrahydrofolate reductase (*MTHFR*) gene in folate metabolism may contribute to epigenetic mechanisms that modify complex gene expression, thus causing autism.* MTHFR* is one of the most important enzymes in the folate pathway. It converts 5,10-methylenetetrahydrofolate to 5-methylenetetrahydrofolate and regulates the intracellular flow of folate. C677T polymorphism in the* MTHFR* gene (A222V, rs1801133) is associated with a decrease in enzymatic activity to 35–70% in homozygotes [18].

To date, there have been some case-control studies of* MTHFR* functional polymorphisms in autism and ASD [14, 15, 17]. Herein we aimed to present our findings on* MTHFR* C677T polymorphism in a total of 98 autistic patients in Turkey.

## 2. Material and Methods

### 2.1. Study Design

*MTHFR* is probably relevant in autism and we wanted to test our study group in Turkey. The study group was collected between May 2009 and May 2014. Patients were diagnosed by using the Diagnostic and Statistical Manual of Mental Disorder, Fourth Edition (DSM-IV) and DSM-V [19, 20]. Exclusion criteria included those who were under the age of 3 and/or diagnosis of genetic or neurological disorder associated with autism. All of the patients had a diagnosis of autism and were followed up at the Department of Child Psychiatry. The case group (*n* = 98) comprised patients with autism and the control group (*n* = 70) comprised healthy children. There were 81 simplex families and 8 multiplex families in the case group. The control group was randomly selected and matched for age and sex for this study. In the study group, there were 71 males and 27 females. Information spanning a minimum of three generations of family history was obtained and simplex families did not reveal further cases of ASDs. All of the cases met the clinical criteria for autism found in the absence of a known etiology with detectable chromosomal defects using the standard karyotyping procedure and other genetic syndromes, that is, Fragile-X syndrome, Rett's syndrome, and Angelman syndrome. The control group consisted of 46 males and 24 females. This study was approved by ethical committee in Erciyes University and informed consent was obtained from all the participants.

### 2.2. DNA Extraction and Genotyping

**Figure 1:**
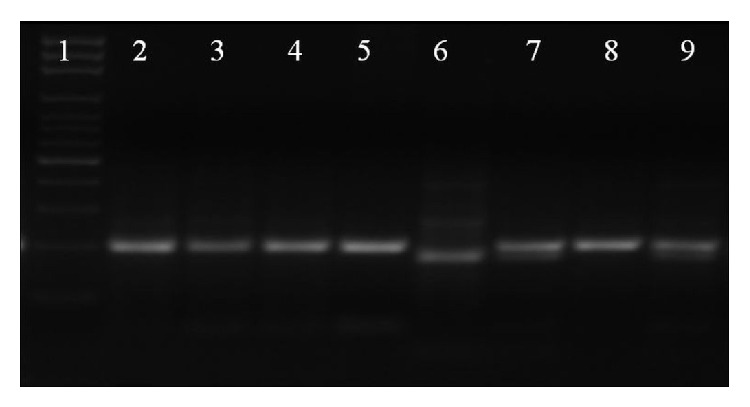
Image of agarose gel electrophoresis after* Hinf 1* restriction enzyme digestion. 1: 100 base pair DNA ladder. 2: PCR product, 3–5, 8: CC genotype, 6: TT genotype, 7, 9: CT genotype.

Two mL blood samples were obtained from patients with autism and from the control group. Genomic DNA was extracted according to the standard protocols of Roche (Roche Magna Pure LC, Germany).* MTHFR* C677T polymorphism (rs1801133) was examined by polymerase chain reaction- (PCR-)restriction fragment length polymorphism (RFLP). PCR fragments were amplified from 20 ng of each DNA sample in 50 *μ*L PCR mix containing 0.5 U/mL* Taq* DNA polymerase, 1.5 mM MgCl_2_, 1X concentration of the buffer, 2.5 mM concentration of deoxynucleotide triphosphate (dNTP), and 10 pmol of each primer. Primer sequences are summarized in Table 1. PCR cycling conditions included initial denaturation at 94°C for 2 min, followed by 40 cycles of 30 sec at 94°C, 30 sec at 62°C, and 30 sec at 72°C, and a final extension step at 72°C for 7 min. One hundred and ninety-eight base pairs of PCR products were then digested overnight at 37°C with* Hinf I* restriction enzyme and checked with 3% agarose gel electrophoresis [21]. Every PCR was accompanied with a negative control without any genomic DNA and a positive control with genomic DNA that is digested completely by the particular restriction enzyme (Figure 1).

### 2.3. Statistical Analysis

To assess the data normality, histogram and *q*-*q* plots were examined; also Shapiro-Wilk's test was applied. To compare the differences between disease groups, Mann-Whitney *U* test was used for continuous variables. Pearson's chi-square analysis was used to determine the relationship between genes in ASD. Odds ratios with 95% confidence intervals were calculated for risk assessment. Analyses were conducted using R 3.1.0 (http://www.r-project.org/) software by considering a *P* value less than 0.05 statistically significant.

## 3. Results

In the study group, there were 71 males (72.4%) and 27 (27.6%) females. The mean age of cases was 6.0 (3.8–8.0). There were 46 (65.7%) male controls and 24 (34.3%) female controls. The mean age of controls was 5.0 (4.0–6.0). There was not any significant difference between the groups for this polymorphism (*P* = 0.237). The heterozygote genotype was associated with 1.3-fold (95% CI: 0.70–2.41) risk for autism and this risk was not statistically significant (Table 2). However,* MTHFR* 677T*-*variant allele frequencies in cases and controls were 29% and 24%, respectively. When we adjusted the analysis according to age effect, we again did not find any statistically significant difference between the groups (*P* = 0.176; 95% CI: 1.37 (0.73–2.58)).

## 4. Discussion

Autism is accepted to have a complex etiology involving both genetic and environmental factors with epigenetic modifications [12, 22, 23]. In this study, we investigated a key gene in folate metabolism. This gene and its polymorphisms have been previously reported to be associated with ASD susceptibility in some case-control studies to date [7, 15, 17]. We could not demonstrate a possible risk for* MTHFR* C677T polymorphism in autism cases.

Liu et al. selected two common polymorphisms (C677T and A1298C) to test simplex and multiplex ASD families because of the association of reduced MTHFR enzyme activity. The T allele was more prevalent in children with an ASD (42.9%) compared to controls (32.3%) and this finding differed significantly from those in the comparison group with a *P* value of 0.0004. However, the frequency of the heterozygous 677CT genotype in the ASD group (47.8%) did not differ from that in the controls (43.2%). The allele and genotype frequencies of the polymorphism in multiplex families were very similar to those in the control group. Finally, they suggested that reduced MTHFR activity is a risk factor for autism only in simplex families [7]. James et al. observed low methionine, low S-adenosyl methionine (SAM)/S-adenosyl homocysteine (SAH) ratio, low cysteine, and low glutathione levels in their study group. They detect significant alteration in homocysteine levels in autism [24]. Paşca et al. studied C677T polymorphism of the* MTHFR* gene in three groups of children diagnosed with autism (*n* = 15), Asperger syndrome (*n* = 5), and PDD-NOS (*n* = 19) and their age- and sex-matched controls (*n* = 25). The results showed a normal distribution of the C677T polymorphism in children with ASDs, but the frequency of the T allele was slightly more prevalent in autistic patients. They, therefore, thought that a possible role for the alterations in one carbon metabolism existed in the pathophysiology of ASDs [25].

In a Chinese Han population, the frequency of the TT genotype of* MTHFR* 677 was significantly higher in children with autism (16.1%) than in controls (8.6%). According to this finding, Guo et al. suggested that* MTHFR* C677T is a risk factor for autistic patients in their population [26]. One hundred and sixty-eight children with a confirmed diagnosis of autism or PDD were investigated by Boris et al. in 2004. Their data demonstrated that 677CT polymorphism, whether in homozygous or heterozygous state, was significantly associated with ASD. They observed an increased frequency of the TT genotype in the autistic children (23%) compared to 11% in the control population [14]. In another study, the case group comprised 151 patients with idiopathic ASDs and 100 healthy controls. The frequency of the T allele was the same (0.38 versus 0.35) between the groups (*P* = 0.77). The genotype distribution did not reveal significant differences between cases and controls (*P* = 0.72) [27].

An interesting study was carried on about behavioral problems in children with autism. Goin-Kochel et al. hypothesized that autistic children who carry the homozygous genotype for the* MTHFR* 677TT would exhibit more behavioral problems and/or more severe problematic behaviors than homozygous wild-type (CC) individuals. Their results did not suggest such a relationship between genotypes for* MTHFR* 677CT and developmental regression. They also showed that four behaviors (current complex body movements, direct gaze, a history of self-injurious behavior, and current overactivity) were more common and problematic among those with a heterozygous state as compared to homozygous wild-type individuals [16]. For Park et al.* MTHFR* 677CT and 1298AC polymorphisms were tested in Korean population. 677CT/1298AC was significantly associated with 2.11-fold increased risk of ASD compared with the combination of 677CC/1298AA genotypes [28]. A meta-analysis was performed by Pu et al. in 2013. They investigated the relationship of the* MTHFR* polymorphisms (C677T and A1298C) and the risk of ASD. Eight case-control studies (1672 patients with ASD and 6760 controls) were included for this meta-analysis. Their results show an association of* MTHFR* C677T polymorphism with increased susceptibility to ASD [29].

In our study genotyping for* MTHFR* 677CT revealed 44 (44.9%) children with the CC genotype, 51 (52%) children with the CT genotype, and 3 (3.1%) children with the TT genotype in cases. We did not detect any homozygous polymorphism in the control group and this result was not significantly different between the groups. T allele frequency was higher in the patients. Our results were in accordance with dos Santos et al.'s study. Thus, contrary to some studies we could not demonstrate an association between this polymorphism and autism. One limitation of this study is the small sample size of autism families. Differences due to the genetic heterogeneity of autism, ethnic variation, recruiting strategies, family types (simplex or multiplex), sample size, and/or other factors that we could account might give these results.

## 5. Conclusion

We suggest replicating the study with larger well-characterized simplex families with nonautistic siblings, to determine the presence or absence of common* MTHFR* SNPs (especially C677T and A1298C). We recommend investigating this risk in a larger sample size with the same diagnostic criteria, same ethnicity and paying great attention to gender distribution to get reliable results in ASD.

Other environmental risk factors should be considered; in particular, the methylation/transsulfation and/or catecholamine-O-methyltransferase (*COMT*) pathways should be investigated in future studies.

## Figures and Tables

**Table 1:** Primer sequences and genotype differences after *Hinf I* enzymatic digestion of *MTHFR* C677T polymorphism.

Primer sequences	CC genotype	CT genotype	TT genotype
F: 5′-TGAAGGAGAAGGTGTCTGCGGGA-3′ R: 5′-AGGACGGTGCGGTGAGAGTG-3′	198 bp	198 bp 175 bp 23 bp	175 bp 23 bp

F: forward, R: reverse.

**Table 2:** Genotype and allelic distribution of *MTHFR* C677T polymorphism in patients with autism and control groups.

	Control (*n* = 70)	Patients (*n* = 98)	*P* value	OR (95% CI)
Female	24 (34.3)	27 (27.6)	0.349	1.00
Male	46 (65.7)	71 (72.4)		1.37 (0.71–2.66)

*MTHFR* C677T				
CC	37 (52.9)	44 (44.9)	0.237	1.00
CT	33 (47.1)	51 (52.0)		1.30 (0.70–2.41)
TT	0 (0.0)	3 (3.1)		—

C allele	0.76	0.71		
T allele	0.24	0.29		

OR: odds ratio; CI: confidence interval.
